# Granulosis rubra nasi from plastic surgery perspective

**DOI:** 10.1093/jscr/rjae547

**Published:** 2024-09-06

**Authors:** Rahaf H Almutairi, Qutaiba N M Shah Mardan, Abdullah A Al Qurashi, Mohamed Amir Mrad

**Affiliations:** College of Medicine, Imam Mohammad Ibn Saud Islamic University, Riyadh, Saudi Arabia; Plastic and Reconstructive Surgery Department, Hamad General Hospital, Hamad Medical Corporation, Doha, Qatar; College of Medicine, King Saud bin Abdulaziz University for Health Sciences, Jeddah, Saudi Arabia; King Abdullah International Medical Research Center, Jeddah, Saudi Arabia; Plastic and Reconstructive Surgery Section, Department of Surgery, King Faisal Specialist Hospital & Research Centre, Riyadh, Saudi Arabia

**Keywords:** granulosis rubra nasi, botulinum toxin A, nose sweating, rhinoplasty

## Abstract

Affecting the eccrine glands, granulosis rubra nasi (GRN) is an inherited disorder that manifests itself as redness, sweating, and papules distributed mainly in the center of the face. It is diagnosed clinically and the cornerstone of management is reassurance and education about the benign nature of the condition. A wide array of treatment modalities has been proposed, such as Botox and corticosteroids injections; however curative measures are yet to be discovered. In this paper, we present a case of a 26-year-old man complaining of chronic nose sweating. The case was successfully managed with Botox injections, though we suggested our theory of undercutting the glands through aggressive defatting as part of open rhinoplasty to the patient; he declined our suggested modality. It remains an untested option and caution should be exercised in high-risk patients to avoid compromising blood supply to the skin and risking skin necrosis.

## Introduction

**Figure 1:**
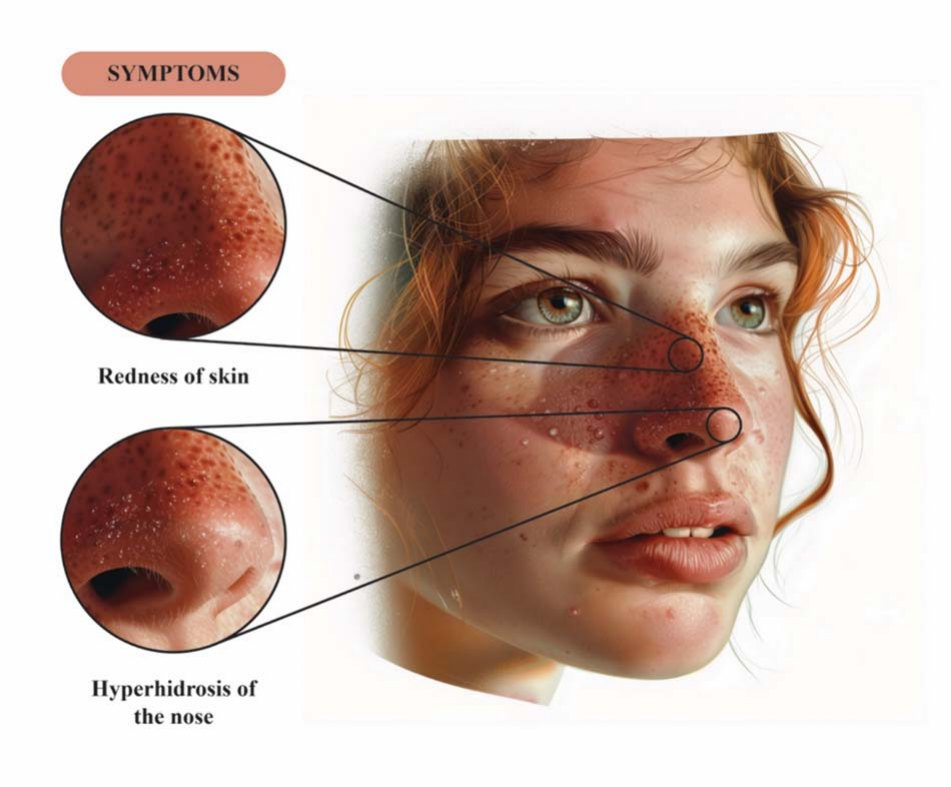
This figure provides an illustration of the clinical features of GRN; beads of sweat seen on an erythematous base over the nose.

Granulosis rubra nasi (GRN) is an autosomal dominant rare disorder of the eccrine glands, also known as “Acne papulo-rosacea of the nose.” [1] This inflammatory condition often first manifests in childhood but can also emerge later in adolescence or adulthood [2]. Hyperhidrosis, erythema, pustules, papules, and vesicles involving the nose are the key characteristics of GRN shown in Fig. 1. It has a chronic course and might disappear spontaneously during puberty [3]. Herein, we report a case of a gentleman who was suspected to have GRN following the STROBE guidelines [3].

## Case presentation

A 26-year-old male presented to the senior author’s clinic complaining of nose sweating for a long time. It was not associated with other complaints, such as redness, pain, or papules; no other areas in the face or body manifested the same complaint. There was no family history. No significant findings were detected during physical examination. He was never diagnosed with GRN and we explained the nature of the condition to him.

Initially, rhinoplasty with aggressive defatting was proposed to the patient; he was informed that his condition was rare and the proposed treatment’s efficacy has not been established. Risks, benefits, and alternative management, Botox injections, were explained to him. He refused rhinoplasty and opted for continuous Botox injections.

Four units of Botox were injected around the tip of the nose, first at his initial visit and then 4 months later with a total follow-up period of 9 months. The concentration used was a bottle of 100 units per vial. Each time, he showed excellent response and was satisfied with the results. The patient refused to disclose his photography for this manuscript.

## Discussion

GRN is a rare inflammatory dermatosis that affects the eccrine glands of the nose, cheeks, and chin [3]. It is believed to be an inherited disease with an autosomal dominant pattern [2]. However, the pathogenesis and etiology are still unexplained [3]. The clinical picture is characterized by hyperhidrosis and diffuse erythema over the central part of the face. This erythema may have sweat droplets embedded within it, giving a moist, glistering appearance to the affected area. Besides, excessive sweating may develop years before the other changes appear.

Due to the rarity of this benign disorder, case reports form the majority of literature around this topic. The diagnosis is typically made through clinical picture. Dilated cutaneous blood and sweat ducts and lymphatic vessels with perivascular lymphocytic infiltrate are observed histologically. The epidermis, pilosebaceous apparatus, and connective tissue remain unaffected [2, 3]. Multiple modalities of GRN treatment have been suggested including oral corticosteroids, topical indomethacin, cryotherapy, and tetracycline [4, 5]. Patient education and reassurance is a crucial part of management. Grazziotin *et al*. [5] reported botulinum toxin A as a treatment for a patient with GRN which resulted in long-term remission. Likewise, Tacrolimus cream was successfully used, as described by Kumar *et al*. [3] a few years ago. Drying lotion such as calamine could also help [2]. Heid *et al*. [6] reported a case of GRN linked with phaeochromocytoma, the condition regressed after surgical removal of the tumor.

**Figure 2:**
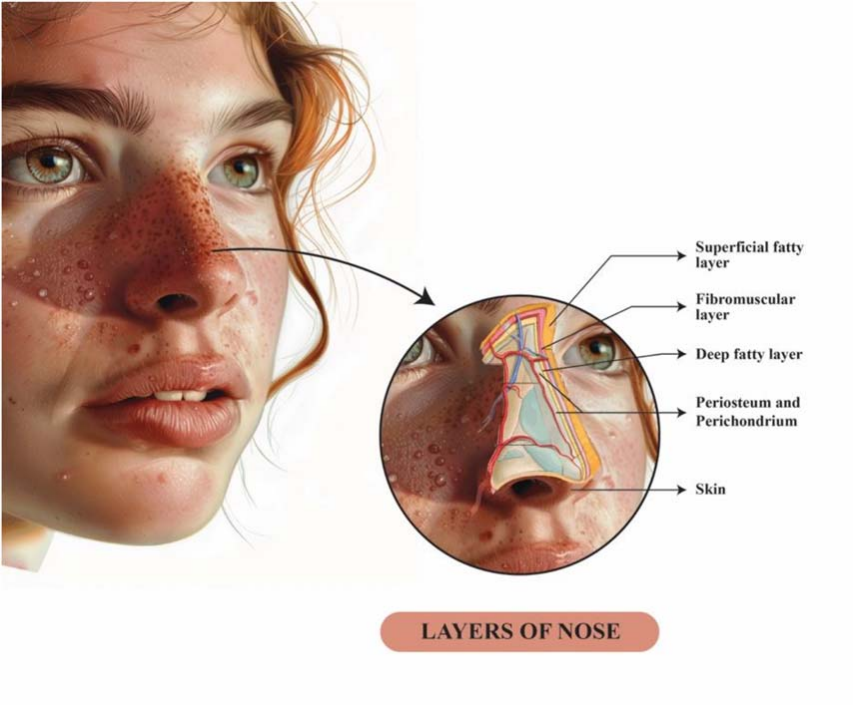
This illustration shows the anatomical soft tissue layers of the nose; during nasal flap elevation in open rhinoplasty, we believe that aggressive defatting and undercutting eccrine glands might cure granulosis rubra nasi.

The eccrine glands are virtually distributed all over the body with higher presence in the axillae, palm of hands, sole of the feet, and face [7]. We hypothesize that by performing aggressive defatting as part of open rhinoplasty, we can remove the sweat glands from the nose which might result in permanent remission of the condition (Fig. 2) unlike Botox injections which require repeated sessions. Furthermore, the affected subject will benefit from the aesthetic byproduct of the procedure. Unfortunately, we were unable to observe the results of our theory as the patient refused to undergo rhinoplasty due to social reasons. Caution should be exercised when aggressively defatting the nose when elevating the skin flap as it may compromise vascularity to the nasal envelope, especially in high-risk patients such as smokers; care should also be taken when splinting the nose to not compromise blood flow [8]. Hurley and Shelley’s [9] paper described the undercutting of subcutaneous tissue to excise sweat gland along with combined limited skin removal from adjacent skin. It subsequently showed a dramatic reduction in hyperhidrosis.

## Conclusion

We reported a case of a gentleman who has been diagnosed clinically with GRN and was successfully managed via regular Botox injection sessions. GRN is a rare inherited disease of eccrine glands. Patients should be educated about the nature of the disease, available treatment modalities, and prognosis. Various approaches of treatment have been reported in the literature, such as Botox injections and topical steroids. We postulate that open rhinoplasty with aggressive defatting of the nasal vault flap might ultimately cure the condition through undercutting the glands. Nonetheless, this should be backed by sufficient evidence prior to adaptation as an approved treatment modality.
